# When one race is not enough: A relay model explains multisensory response times

**DOI:** 10.1371/journal.pcbi.1013320

**Published:** 2026-05-26

**Authors:** Kalvin Roberts, Thomas U. Otto

**Affiliations:** School of Psychology and Neuroscience, University of St. Andrews, St. Andrews, United Kingdom; Durham University, UNITED KINGDOM OF GREAT BRITAIN AND NORTHERN IRELAND

## Abstract

Humans typically respond faster to multisensory signals than to their unisensory components, a phenomenon known as the redundant signal effect (RSE). One of the earliest and most influential accounts, the race model, attributes the RSE to statistical facilitation, which arises from parallel, independent processing across sensory modalities. While this model captures some key features of the RSE, it frequently underestimates the observed speed-up leading to violations of the race model inequality (RMI), a benchmark used to test the model’s validity. To reconcile this discrepancy, we introduce the relay model, a minimal extension of the race architecture that incorporates cross-modal initiation. In this model, responses result from two sequential race processes, allowing a signal in one modality to initiate the onset of perceptual decision processing in another. This structure retains statistical facilitation as a core principle while introducing a single free model parameter that partitions unisensory processing into gating and decision stages. Through simulations and fits to foundational empirical datasets, we show that the relay model captures both the magnitude and distributional shape of the RSE, including RMI violations. It also accounts for changes in the RSE under asynchronous stimulus onsets and manipulations of signal intensity, which are critical tests in multisensory research. By extending the classical race model with minimal added complexity, the relay model offers a mechanistically explicit and biologically plausible framework for explaining the dynamics of multisensory decision-making.

## Author summary

How does the brain combine information from different senses, like hearing and seeing? For decades, researchers have found that people respond faster when signals from multiple senses occur together than when signals come from a single sense alone. Two main ideas have been proposed to explain this effect: one suggests that the senses operate independently, racing to trigger a response, while the other suggests that they cooperate to enhance processing. We here introduce a new computational account, the relay model, that bridges these ideas. In this model, perceptual decisions unfold in two stages. A first gating stage detects incoming signals and initiates a second decision stage that leads to a behavioural response. Importantly, detection in one sense can initiate decision-making in another. This relay-like mechanism allows both competition and cooperation between the senses. By capturing key findings from classic experiments, the model offers a new perspective on how the brain combines multisensory information to guide behaviour.

## Introduction

Perceptual decisions are rarely based on information from a single sense. Instead, the brain continuously uses inputs from multiple modalities to guide rapid and adaptive behaviour. This multisensory integration enhances accuracy and accelerates response times (RTs). For instance, if a person both sees an oncoming bicycle and hears its bell, they are likely to react more quickly than if only one of those signals were available. Understanding the computational mechanisms underlying multisensory integration is essential for building models that capture how the brain makes fast and reliable decisions in dynamic environments.

One of the most widely used paradigms to study the benefits of multisensory processing is the redundant signals paradigm [1–4]. In this task, participants are instructed to respond with the same action to different target signals, such as an auditory signal, a visual signal, or both. The critical feature is that signals in the combined condition are redundant, either alone is sufficient for a correct response. Thus, the task effectively implements a logical OR operation [5–7]. The key finding is that responses to redundant signals are, on average, faster than responses to single signals, a phenomenon known as the redundant signals effect (RSE). The effect has been replicated across a wide range of multisensory pairings (e.g., [8–14]), within single modalities (e.g., [15–20]), across populations (e.g., [21–25]), and under varying task instructions (e.g., [26–30]). This robustness makes the RSE a valuable tool for probing the computational principles of multisensory decision-making.

To understand the mechanisms behind this speed-up, it is useful to first consider how unisensory decisions are modelled. Computational and neurophysiological studies suggest that a key component is the accumulation of sensory evidence over time [31–33]. Specialised neurons modulate their firing in response to stimuli, but because of intrinsic noise, evidence must be integrated over time to improve reliability. Once a decision threshold is reached, a behavioural response is triggered. This process gives rise to typical RT distributions, such as the positively skewed inverse Gaussian (IG) [34,35], and has proven effective in capturing unisensory decision dynamics [36]. The central question, then, is: how can these models be extended to multisensory decisions?

**Fig 1:**
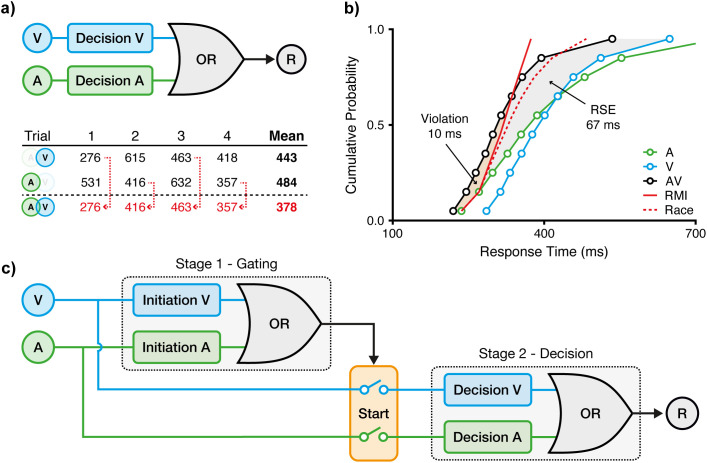
Race models in multisensory decision-making. **a)** Raab’s race model. Auditory (A) and visual (V) signals are processed by two parallel decision units, coupled via a logical OR gate to trigger a response (R). The model predicts faster responses for redundant audiovisual (AV) signals through statistical facilitation, as illustrated by exemplary response times (RTs). On each AV trial, a response is determined by the faster of the two units. Averaging RTs across trials reveals a speed-up of responses for AV compared to unisensory signals. **b)** Miller’s race model inequality (RMI) test. The cumulative RT distribution with redundant AV signals is located to the left of the unisensory RT distributions (A, V), reflecting the redundant signals effect (RSE, grey area). The observed RSE exceeds the prediction of Raab’s race model, including significant violation of the RMI (orange area), indicating a processing interaction between audition and vision. Data replotted from [4]. **c)** Relay model. To reconcile Raab’s race model with RMI violations, we propose a two-stage model comprising two sequential race processes. As an audiovisual processing interaction, the first gating stage provides a start signal for the second decision stage. The model allows for cross-modal initiation; for instance, a fast gating in vision can initiate decision process in both modalities. The second decision stage operates analogously to Raab’s race model to trigger a response.


A milestone answer was offered by Raab [5], who proposed the classical race model (Fig 1a). Here, two independent evidence accumulation units operate in parallel, each detecting one of the sensory signals. These units are linked by a logical OR gate, meaning that a response is triggered as soon as one or the other unit reaches its decision threshold. When both signals are presented, statistical facilitation occurs: if one process is slower on any given trial, the other may trigger the response first, thereby reducing average RTs (see example trials in Fig 1a). With audio-visual signals (AV), the mechanism of the race model is captured by selecting the faster of two unisensory RTs (for A and V, respectively):

$$\begin{array}{c} {RT}_{AV}=\text{min}\left({RT}_{A},\;{RT}_{V}\right) \end{array}$$ (1)

Consequently, the cumulative distribution function (CDF) for RTs in the redundant condition follows probability summation, where the chance of responding by time *t* increases by having two independent chances to respond:


$$\begin{array}{c} F_{AV}(\text{t})=F_{A}(\text{t})+F_{V}(t)-F_{A}(\text{t})\times \;F_{V}(\text{t}) \end{array}$$ (2)

Here, F_AV_(t) is the cumulative probability of responding at time t to both signals, and F_A_(t) and F_V_(t) are the unisensory RT distributions. Thus, race models provide parameter-free predictions of the RSE based on unisensory data, offering concrete and testable predictions about the expected magnitude of the RSE and the conditions under which it should be most pronounced.

Building on probability summation in the race model, two key principles to predict the expected speed-up emerge [37]. First, the *principle of congruent effectiveness* predicts that the RSE is largest when unisensory RT distributions overlap. This overlap can be optimised by introducing a temporal delay between signals, known as stimulus onset asynchrony (SOA), which can offset inherent RT differences. For instance, if auditory responses are on average 30 ms faster, delaying the auditory relative to the visual signal by 30 ms aligns the two unisensory RT distributions and maximises the RSE (e.g., [2,37,38]). Second, the *variability rule* states that the RSE is driven by the variability of RTs in unisensory conditions, which can be modulated for example through signal intensity. As weaker signals tend to produce greater RT variability, the RSE is expected, and has been shown, to increase under such conditions (e.g., [37,39]). Together, these findings show that the race model captures key patterns of multisensory facilitation, reinforcing its value as a simple yet powerful explanatory framework.

Despite their predictive power, race models have clear limitations. As a second milestone, Miller [4] demonstrated that the RSE often exceeds what can be explained by probability summation alone. Allowing for a maximally negative correlation between unisensory processes [40], the probability of responding to redundant signals cannot exceed the sum of the individual unisensory probabilities:


$$\begin{array}{c} F_{AV}(\text{t})\leq F_{A}(\text{t})+F_{V}(t) \end{array}$$ (3)

This so-called race model inequality (RMI) provides a testable boundary for race model predictions (Fig 1b). Empirical RMI violations demonstrate that the observed RSE cannot be attributed to the race model alone, implying some interaction between sensory channels. Given the consistent reports of RMI violations, many researchers have moved away from race accounts (with some notable exceptions, [11,41]) and toward *single-threshold* accounts. In these models, sensory evidence from two (or more) modalities may be pooled in a single decision process to reach a shared threshold [e.g., 4, 42]. These pooling models are commonly referred to as coactivation models, although the term has not always been used consistently in the literature [43].

Importantly, the RMI, and classical race models in general, implicitly assume a single-stage architecture: two isolated, parallel accumulation processes leading directly to the same response (for comprehensive discussions of the underlying assumptions, see [7,44,45]). This assumption is analytically convenient but biologically simplistic. The pathway from sensory transduction to motor execution is unlikely to consist of a single step. Instead, information likely traverses multiple sequential processing stages [46,47]. At a purely agnostic level, these stages represent discrete processing epochs, regardless of their specific cognitive or neural nature. In principle, there could be many such stages, encompassing sensory encoding, feature extraction, and motor preparation [48]. The race model’s limitation to a single processing stage is particularly relevant in a multisensory context, where interactions between modalities can occur at multiple levels [49–51]. As soon as the decision process includes sequential stages with the potential for crossmodal interactions, the foundational independence assumptions of the RMI framework no longer hold [52]. This raises a critical question: how can multisensory decision-making models be extended to capture biologically plausible multi-stage dynamics?

In unisensory decision-making, such models are increasingly prevalent. One particularly compelling candidate for the cognitive nature of these distinct stages posits an initial “gating” or selection process followed by a subsequent “decision” process involving evidence accumulation. For example, Carpenter et al. [53] proposed a two-stage architecture in which early detection units trigger a later accumulation unit (see also [47,54–57]). In these models, the initial gating stage determines when evidence accumulation begins, preventing the system from integrating noise before relevant sensory input is available [47,58]. Without such gating, accumulation could start before stimulus onset, leading to random responses, increased RT variability, and unreliable confidence estimates. Biologically, gating may arise through sensory onset signals, internal go cues, or inhibitory dynamics that suppress pre-stimulus noise [47,58]. In modelling terms, this is often implemented as a gate function or a reset of initial conditions. Separating gating from evidence accumulation therefore provides a highly plausible mechanism that supports both computational stability and biological realism.

In multistage frameworks, the total RT is expressed as the sum of processing times associated with these individual stages (e.g., RT_A_ = T_A1_ + T_A2_ for an auditory stimulus; RT_V_ = T_v1_ + T_V2_ for a visual stimulus). This additive logic is also reflected in standard models of perceptual decision making, such as the drift diffusion model [48], where the total RT is partitioned into a core decision process, which incorporates evidence accumulation, and a residual non-decision time, which subsumes early sensory encoding and late motor execution. While such multi-stage frameworks are well established in unisensory contexts, their extension to multisensory decision-making remains comparatively underexplored (but see [42,59]).

In this study, we develop and test an extension of the race model to address this gap in research on multisensory decision making. The model consists of two sequential stages: a first gating stage that detects incoming signals which initiates a second decision stage that leads to a behavioural response (Fig 1c). Critically, each stage processes auditory and visual signals in parallel, as in the standard race model (Equation 1). As a result, the total multisensory RT is given by the sum of two race processes:


$$\begin{array}{c} RT_{AV}=min\left(T_{A\text{1}},\;{\;T}_{V\text{1}}\right)+\;min\left(T_{A\text{2}},\;{\;T}_{V\text{2}}\right) \end{array}$$ (4)

At the first gating stage, auditory and visual processes race to initiate the decision stage. The modality that wins this race effectively “passes the baton” to the next stage, which motivates the model’s metaphoric name: the *relay model*. Crucially, initiation at the first gating stage is *non-competitive*. The winner of this first race (e.g., a fast visual signal, $T_{V\text{1}}$) does not suppress the other modality but instead initiates the second stage of parallel decision processes for both modalities ($T_{A\text{2}}$ and $T_{V\text{2}}$). Because these processes are assumed to be statistically independent, the outcome of the first stage does not deterministically dictate the final response. Due to trial-by-trial variability in evidence accumulation, the initially slower modality can still win the second stage.

A key property of the relay architecture is that it naturally produces RT distributions that can exceed the predictions of standard race models. Because the decision process unfolds across race processes at sequential stages, the effective RT reflects both the faster gating signal and the outcome of a subsequent decision race. Crucially, this structure introduces cross-modal dependencies, which violate the independence assumptions underlying the RMI. As a result, we show below that RMI violations, previously unexplained by the race model, emerge naturally from the relay architecture. In doing so, the model provides a simple and biologically plausible alternative to pooling or coactivation accounts. Beyond reproducing the magnitude of the RSE, we demonstrate that the relay model inherently captures how decision dynamics change with temporal asynchrony or manipulations of signal strength. Thus, by reconciling the milestone contributions by Raab [5] and Miller [4], the model offers a computationally grounded framework that reflects the staged nature of perceptual decisions in multisensory contexts.

## Results

### The relay model explains multisensory processing dynamics

To evaluate the plausibility of the relay model, we tested whether its predictions align with empirical RTs reported in Miller’s benchmark dataset (data estimated from Figure 1 in [4]; see Methods). As a key advancement in understanding multisensory processing, the model assumes that perceptual decision processes comprise latent sequential processing stages. For example, an auditory RT is modelled as the sum of two stages (e.g., RT_A_ = T_A1_ + T_A2_).

To constrain the model, we fitted IG distributions to the overall RTs in unisensory auditory and visual conditions (Table 1; see Methods). This distribution is characterised by two parameters: the mean (μ) and the shape parameter (λ). We estimated these parameters by minimising the root mean squared error (RMSE) between empirical and model CDFs. These unisensory parameters were held fixed to constrain the modelling of the multisensory condition.

**Table 1:** Data summary and best-fitting unisensory distribution parameters.

Study	Experiment	N	Trials (total)	Modality	μ (ms)	λ (ms)
Miller (1982)	Exp. 1	74	1480	A	399	3272
				V	402	7309
Miller (1986)	B.D.	1	400	A	231	3931
				V	348	4979
Otto et al. (2013)	SOA	10	1600	A	376 (23)	8619 (738)
				V	404 (24)	14225 (2138)
Otto et al. (2013)	Intensity	20	2000	A_S_	412 (11)	13925 (1064)
				A_M_	457 (11)	11711 (949)
				A_W_	508 (11)	7888 (595)
				V_S_	431 (10)	15486 (1450)
				V_M_	515 (13)	9534 (630)

*Note*: Miller (1982) data were fitted to group-average RTs. Otto et al. (2013) data were fitted per participant; values show cross-participant mean (SEM). μ and λ are the mean and shape of the IG distribution.

**Fig 2:**
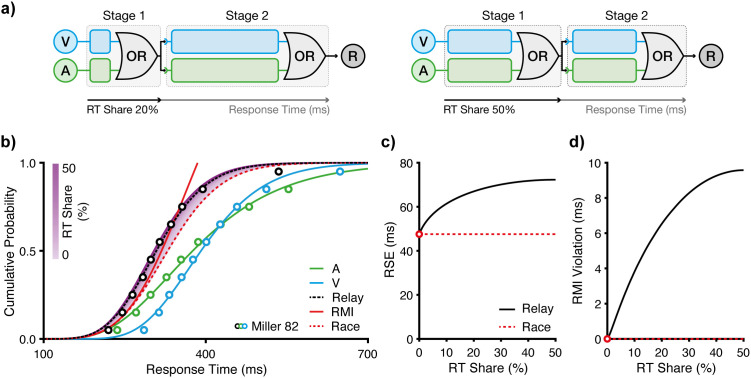
Predictions of the relay model. **a)** RT share. In the relay model, the RT share denotes the proportion (%) of the overall RT attributed to the first stage: 20% (left) and 50% (right). While these schematic diagrams illustrate this partitioning for a single trial, at the distribution level, the RT share parameter specifies how the entire RT distribution (both its mean and variability) is divided into two sequential stages for each modality. The predicted multisensory RT distribution is then formed by summing the outcomes of the independent races at each stage. Schematic diagrams correspond to the model architecture introduced in Fig 1c. **b)** Modelling based on data from Miller [4]. The relay model is constrained by the RT distribution for auditory (A) and visual (V) signals (Table 1). The shaded region depicts relay model predictions for RT shares ranging from 0% to 50%. The empirical RT distribution for redundant audiovisual (AV) signals is best fit with an RT share of 22.6%. Circles represent the same data shown in Fig 1b. **c)** RSE and **d)** RMI violations as a function of RT share. The relay model predicts RSEs that exceed those of Raab’s race model, including systematic RMI violations. Predictions decline symmetrically for RT shares above 50% (e.g., RT shares of 20% and 80% yield identical predictions).

To partition the overall RT into stage-specific latent components, we introduce the RT share parameter (Fig 2a). It expresses the contribution of the first stage as a proportion of the overall RT. For instance, if RT_A_ is on average 400 ms and the first stage (T_A1_) lasts 80 ms, then T_A1_ contributes 20% of the total RT. The variability of RTs across trials is partitioned analogously.

We formalised this partitioning approach using the IG distributions fitted to the unisensory conditions. Specifically, we exploit a property of the IG distribution that allows for mathematically consistent partitioning, whereby the overall RT can be split into sequential stages using the RT share parameter, while each latent stage remains IG-distributed. This property preserves analytical tractability, allowing us to model the latent processing stages without resorting to numerical simulations. For simplicity, we used a single RT share parameter for both modalities; however, the model can be extended to support distinct RT shares per modality, which would reflect differences in sensory latencies. The RT share thus expresses the relative contribution of the first of two IG-modelled processing stages, thereby shaping how the system transitions across sensory modalities and stages.

To predict RTs in multisensory conditions, the relay model assumes a non-competitive relay architecture (Fig 1c; Eq 4). During the first gating stage, unisensory processes (e.g., T_A1_, T_V1_) race to completion. The winner then initiates the second decision stage, triggering a race between the second stage processes (e.g., T_A2_, T_V2_). The winner of this second race determines the behavioural response. Crucially, this architecture enables cross-modal initiation: for example, a visual stage 1 winner can trigger an auditory stage 2 process. This feature distinguishes the relay model from the classical race model.

To examine how the RT share affects model behaviour, we simulated the relay model across RT share values from 0% to 50% (Fig 2b). As the two stages add up to 100%, this range encompasses the full dynamic range (values greater than 50% are symmetric). At 0% RT share, the relay model reduces to the classical race model (Fig 1a; Eq 2), which predicts an RSE of 48 ms. In contrast, the relay model predicts an amplification of the RSE up to 72 ms (1.5 × the race model) at 50% RT share (Fig 2c). The race model also predicts no violations of the RMI, but with an RT share of 50%, the relay model predicts RMI violations in the order of 10 ms (Fig 2d). Thus, the RT share, as a single interpretable parameter that implements the relay architecture, enables the model to capture the key effects observed in human multisensory behaviour.

To evaluate the model’s fit to empirical data, we estimated the RT share by minimising the RMSE between empirical and predicted multisensory CDFs (Fig 1b). For parsimony, we again constrained auditory and visual RT shares to be equal. The best-fitting RT share was 22.6% (RMSE = 0.023), corresponding to first-stage components of 89.9 ms (SD = 31.4 ms) for audition, and 90.6 ms (SD = 21.3 ms) for vision. To compare the model fit to the empirical data, we computed RSE and RMI violations at the ten quantiles (as opposed to over the whole distribution as in Fig 2c, d). The best-fitting model predicted an RSE of 68 ms (empirical: 67 ms) and RMI violations of 8 ms (empirical: 10 ms). The slight attenuation of the predicted violation is most likely attributable to the constraints of the unisensory fits; specifically, the IG distribution does not perfectly capture the extreme fast tail of the empirical RT distributions, where RMI violations are most prominent (see fastest quantiles in Fig 2b). Yet, overall, the model captures the distributional shape of the AV condition with high accuracy.

To further validate the relay model, we compared its performance against a “shifted” race model, an alternative ad-hoc formulation that can also be used to explain RMI violations with a single free parameter. In this model, the classical race model prediction is simply shifted in time by a free parameter, δ, effectively translating the entire distribution to faster RTs. Conceptually, the shift may indicate a non-specific alerting effect, where the presence of multisensory signals elevates baseline arousal and accelerates RTs. The shifted race can account for RMI violations in Miller’s data [4] with a shift of 19.2 ms. However, it provides a poorer fit to the dataset (RMSE = 0.032) compared to the relay model (RMSE = 0.023), which thus shows better explanatory power.

In summary, with only one additional degree of freedom beyond the fits obtained from unisensory conditions, the relay model successfully reproduces a hallmark signature of multisensory integration: larger RSEs than predicted by the race model, including RMI violations. These findings demonstrate that a simple relay architecture offers a parsimonious and powerful framework for modelling the dynamics of multisensory processing.

### Predictions of multi-stage relay models

**Fig 3:**
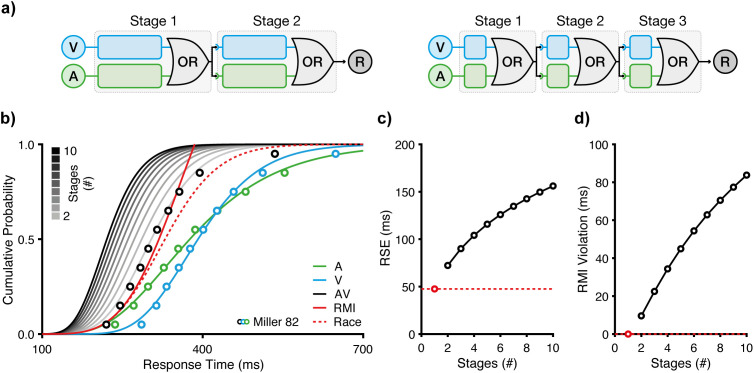
Multi-stage relay models. **a)** Number of stages. Relay models with two and three sequential stages are shown. Each stage passes a start signal to the next, with the overall RT evenly distributed across stages. The two-stage schematic diagram corresponds to the architecture introduced in Fig 1c. **b)** Modelling based on data from Miller [4]. Greyscale lines depict predicted cumulative RT distributions for increasing numbers of stages, from two up to ten. Circles represent the same data shown in Fig 1b. **c)** RSE and **d)** RMI violations as a function of the number of relay stages. Relay models with three or more stages predict RSEs and RMI violations that far exceed empirical observations. Predictions from Raab’s race model (one stage) are indicated by red circles for reference.

While our initial analyses focused on a two-stage relay architecture, the model is not inherently restricted to two processing stages. In principle, multisensory decision-making could involve three or more stages, with each stage comprising a race between modalities (Fig 3a). This raises a key question: how many relay stages account for empirical multisensory RTs best?

To address this, we simulated relay architectures with one to ten stages, using the same unisensory IG fits to constrain each model (Fig 3b). In every simulation, the total RT was evenly divided across N stages, such that each stage was allocated 1/N of the total RT. Accordingly, the RT share was held fixed at this proportion (i.e., not optimised). This design enabled us to assess how increasing the stage count affects predicted RSEs and RMI violations, while holding model complexity and parameterisation constant.

Simulation results show that both predicted RSEs and RMI violations increase with stage count, but with diminishing returns (Fig 3c,d). The most substantial gain occurs between the race model and the two-stage relay model. Adding more stages yielded progressively smaller gains. Crucially, higher stage counts led to increasingly exaggerated predictions. For example, the ten-stage model predicted an RSE of 156 ms and RMI violations of 84 ms, which far exceed empirically observed values.

To evaluate these architectures quantitatively, we compared predicted to empirical multisensory CDFs (Fig 3b). Because the RT share was fixed (rather than optimised) and all models shared identical unisensory parameters, this provided a fair, complexity-controlled comparison. The two-stage relay model emerged as the best configuration, yielding the lowest RMSE (RMSE = 0.030), more than twice as accurate as the one-stage race model (RMSE = 0.085). Adding further stages degraded model performance (e.g., three-stage RMSE = 0.102; ten-stage RMSE = 0.399), consistent with their overestimation of RSE and RMI effects.

Crucially, the assumption of evenly distributed stages is not a strict requirement of the relay architecture. In a relay with more than two stages, the RT shares can be asymmetric. Indeed, perceptual decision-making can be thought of as asymmetric stages of sensory encoding, evidence accumulation, and motor execution, where the vast majority of RT is dedicated to the accumulation of evidence [32,48,60]. For instance, an alternative three-stage relay configuration could consist of a brief initial stage (10%), a longer middle stage (80%), and a brief final stage (10%). This 10-80-10 distribution yields predictions much closer to the two-stage model (RMSE = 0.039). Therefore, while a two-stage relay is sufficient to capture the current data, there are likely situations where asymmetric, multi-stage relays can be modelled successfully.

In summary, while the relay architecture can, in principle, support an arbitrary number of stages, the two-stage configuration strikes the optimal balance. It captures observed multisensory enhancements without inflating predictions beyond empirical observations, though unequal multi-stage models remain a viable architecture.

### Temporal dynamics of multisensory decisions

**Fig 4:**
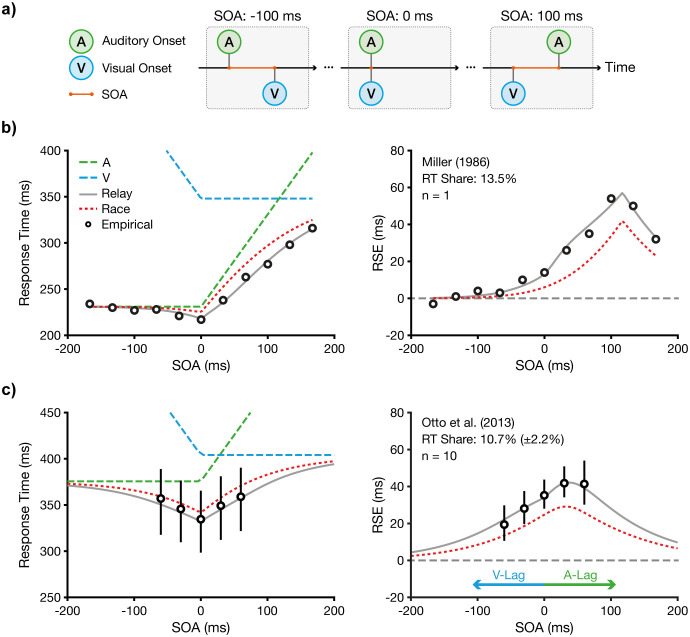
Temporal dynamics in multisensory processing. **a)** Stimulus onset synchrony (SOA). Negative SOAs represent visual lags relative to auditory signal onset (left). An SOA of 0 ms represents synchrony between the auditory and visual onsets (centre). Positive SOAs represent auditory lags relative to visual onset (right). **b)** Modelling based on data from Miller [38] (n = 1). Circles show mean RTs with redundant signals (left) and the RSE (right) as a function of SOA. Dashed lines represent unisensory RTs, adjusted for SOA (e.g., the SOA is added to auditory RTs for positive SOAs). Red lines show predictions from Raab’s race model and grey lines the relay model, which fitted the empirical RTs best with an RT share of 13.5%. **c)** Identical analysis and modelling based on data from Otto et al. [37] (n = 10). The relay model fitted the empirical RTs best with a mean RT share of 10.7 ± 2.2% (model fitting was performed at the participant level). Data points show group averages; error bars represent bootstrapped 95% confidence intervals.

Having established that the two-stage relay model captures both RSE and RMI violations under standard conditions, we next examined its ability to capture multisensory behaviour under conditions of temporal asynchrony. Specifically, we simulated SOA conditions, in which one sensory signal precedes the other (Fig 4a). As a hallmark manipulation in multisensory research, SOA manipulations provide a stringent test of multisensory models by probing how response dynamics adapt when stimuli are no longer presented simultaneously [e.g., 38,61,62].

We used the benchmark dataset from Miller [38], which systematically varied SOAs between auditory and visual signals from -167 ms (auditory lead) to +167 ms (visual lead) in 33 ms increments (Fig 4b). This dataset exhibits two characteristic features. First, because any deviation from synchrony introduces a delay, the fastest multisensory responses occur under synchronous conditions (SOA = 0 ms). Second, the largest RSE is observed when the SOA compensates for the difference in unisensory RTs, a phenomenon introduced earlier as the principle of congruent effectiveness [37]. In Miller’s data, visual RTs were ~100 ms slower than auditory RTs. Accordingly, the largest RSE was observed when the auditory signal lagged by ~100 ms. While the classical race model captures these key characteristics, it systematically underestimates RSE magnitudes across SOA conditions.

To accommodate temporal asynchrony in the relay model, we implemented a simple model extension. Since evidence accumulation cannot begin prior to stimulus onset, we incorporated a temporal delay into the processing of the lagging signal, equal to the experimentally defined SOA. Thus, at stage 1, processing for each modality begins with its signal onset. For example, if the auditory signal is delayed by τ, the stage 1 completion time becomes $T_{\text{1}}=min\left(T_{A\text{1}}+\tau ,\;{\;T}_{V\text{1}}\right)$. Critically, we retained the relay model’s core feature of cross-modal initiation: stage 2 processing is initiated by the first modality to complete stage 1. If stage 2 is initiated before the delayed signal is physically present, processing of that modality is further delayed until its onset. In the model, we account for this by adding a corresponding residual delay at stage 2. This mechanism becomes increasingly important at larger SOAs, where the lagging modality may be partially or fully excluded from processing. When the SOA exceeds the combined duration of both stages, the model response effectively reduces to the unisensory condition of the leading signal.

We tested whether this model extension could fully reproduce the empirical data. First, we constrained the model by fitting its unisensory parameters (Table 1). With these parameters fixed, we optimised the model’s single free parameter, the RT share, by minimising the RMSE between predicted and observed RSEs across SOA levels. The best-fitting RT share was 13.5% (RMSE = 2.5 ms), corresponding to first-stage durations of 31.2 ms (SD = 7.6 ms) for auditory, and 47.0 ms (SD = 12.4 ms) for vision. Overall, the best-fitting model produced an excellent fit to all empirical data points.

As it is difficult to generalise model performance from a single participant, we applied the same analysis to a second dataset from Otto et al. [37], which employed a redundant signal paradigm with 10 participants (Fig 4c). Here, the SOA varied from -60 ms (auditory lead) to +60 ms (visual lead) in 30 ms increments. Consistent with the previous analysis, the fastest multisensory RTs occurred under the synchronous condition. Moreover, the largest RSE was observed when the SOA compensated for the difference in unisensory RTs. Here, visual RTs were on average ~30 ms slower than auditory RTs. Accordingly, the largest RSE occurred for an SOA of 30 ms. Again, the race model predicted these temporal dynamics but failed to capture the magnitude of the empirical RSE.

We employed the same model analysis as above, with all fitting performed using RT distributions at the participant level. As a first step, we constrained the model with the IG parameters estimated in unisensory conditions using Quantile Maximum Probability Estimation (QMPE; [63,64]; Table 1). We then fitted the relay model with its single free parameter, RT share, to the RT quantiles across the six multisensory SOA conditions. The mean estimated RT share was 10.7 ± 2.2%, with a mean Negative Log-Likelihood (NLL) of 368.1. A sign-flipping permutation test (10,000 iterations) confirmed that this RT share was significantly different from zero (p = 0.002). Crucially, with only this single degree of freedom at the multisensory level, the model predicted the observed RSE across all SOA conditions, always falling within the bootstrapped 95% confidence intervals.

Thus, the relay model successfully captured both qualitative and quantitative features of the datasets from Miller [38] and Otto et al. [37]. It predicted the fastest RTs at synchronous onset, identified the SOA that maximised the RSE, and closely matched the RSE magnitudes across all SOA conditions (Fig 4b,c). By incorporating a two-stage architecture that allows for cross-modal initiation, the relay model offers a mechanistically grounded account of how temporal asynchrony shapes multisensory decision-making, thereby seamlessly transitioning between unisensory and multisensory conditions.

### Manipulation of signal strength

To further evaluate the relay model, we analysed data from a redundant signals paradigm with varying signal strength [37]. Such manipulations are established tools known to systematically modulate both the mean and the variability of unisensory RTs [65]. In general, stronger signals yield faster and more consistent RTs, whereas weaker signals produce slower and more variable responses. Critically, race models predict that signal strength manipulations have a similar effect on the RSE as SOA manipulations, because unisensory RTs need to be equally effective to maximise the RSE (principle of congruent effectiveness). In addition, such manipulations are also expected to affect the RSE because, according to race models, it is the variability of unisensory RTs that drives the effect (variability rule). Consequently, it is not surprising that signal strength manipulations also have strong empirical effects on the RSE [37,61,66], making them a stringent test of the model’s predictive power.

**Fig 5:**
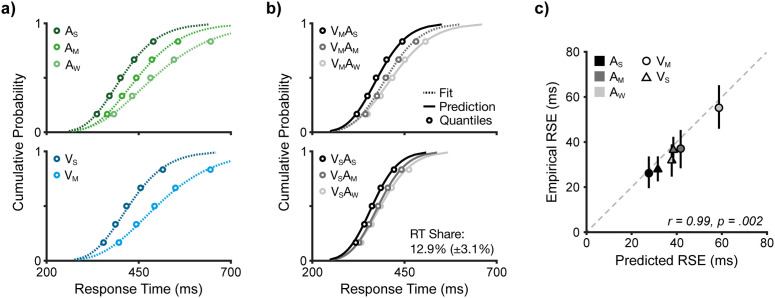
Signal strength manipulation. **a)** Unisensory RT distributions. Signal strength systematically modulated unisensory RTs, with strong effects on both mean and variability. Empirical data are represented by quantiles (circles) and IG fits (dashed lines; for best-fitting parameter values, see Table 1). **b)** Multisensory RT distributions. Signal strength also modulated multisensory RTs. The relay model was fitted in the condition with both modalities at the medium level (dashed line). The best-fitting estimate of the RT share parameter was 12.9% ± 3.1%. Keeping this parameter value fixed, the relay model predicted the RT distributions for the remaining five combinations of signal strength (solid lines). **c)** RSE across signal strength combinations. A near-perfect correspondence was found between predicted and empirical RSEs. Data taken from [37].

In the dataset by Otto et al. [37], signal strength was manipulated in both modalities (Fig 5a). We analysed three auditory levels (weak, medium, strong; A_W_, A_M_, A_S_) and two visual levels (medium, strong; V_M_, V_S_). The dataset also included a third, weak visual signal, however, performance in this condition was not at ceiling and thus cannot be used in the modelling approach employed here, which assumes ceiling performance (see Methods). The signal strength manipulations produced substantial effects on unisensory RTs, with changes in the mean up to ~100 ms and in the variability up to a factor of two. Using a model fitting strategy at the participant level, we estimated the unisensory parameters using QMPE [63,64]. For each signal strength condition, we fitted the two unisensory parameters, yielding a total of ten parameters across five RT distributions (Table 1). As in the approaches above, these parameters were held fixed and used to constrain the modelling of the multisensory conditions.

The unisensory conditions were combined using a 3 x 2 factorial design, yielding six multisensory conditions ranging from the weakest (A_W_V_M_) to the strongest combination (A_S_V_S_; Fig 5b). In a first step, we fitted the relay model with a single free parameter (RT share) to the RT quantiles of the multisensory condition with both modalities at the medium level (A_M_V_M_). The mean estimated RT share was 12.9% ± 3.1% (mean NLL = 93.4), which was significantly different from zero (permutation test, *p* < .001). In a second step, we generated model predictions for the remaining five multisensory conditions. Crucially, these predictions were derived with no additional free parameters, relying solely on the best-fitting RT share from the medium condition (A_M_V_M_) and the IG parameters in the underlying unisensory conditions (Table 1). The model predicted all empirical distributions with high fidelity.

To summarise this model analysis, we examined the modulation of the RSE for the different signal strength combinations (Fig 5c). In line with the strong modulation of unisensory RTs, the empirical RSE was significantly modulated by approximately a factor of two, ranging from 28 ± 3 ms (A_S_V_S_) to 55 ± 5 ms (A_W_V_M_; permutation test, *p* < .001). This modulation was accurately predicted by the relay model (as fitted above). A correlation analysis revealed a near-perfect correspondence between the empirical and predicted RSEs (*r* = .99, permutation test, *p* = .002; Fig 5c). This result demonstrates that a single, invariant relay mechanism is sufficient to explain the complex pattern of facilitation across varying levels of signal strength without requiring additional parameter adjustments, highlighting its strong explanatory power across a second key experimental manipulation.

## Discussion

The robust speed-up of responses observed with multi- compared to unisensory signals provides a critical window into the computational strategies underlying perceptual decision-making. The RSE not only reflects a prominent performance advantage but also places strong constraints on models that explain how the brain combines information across modalities. As one of the earliest and most influential frameworks, the race model [5] proposes that responses are triggered by the faster unisensory signal to reach its decision threshold, assuming that modalities operate independently and in parallel (Fig 1a). While this model accounts for the RSE through statistical facilitation, it typically underestimates the observed behavioural enhancement. Most notably, it imposes the RMI as an upper bound on the RSE, which is frequently violated empirically (as demonstrated in numerous follow-ups to [4]; Fig 1b). These systematic violations strongly argue against purely parallel architectures and have led many to favour explanations in which multisensory inputs are pooled or integrated into a single decision process (e.g., [67–70]).

To reconcile the conceptual parsimony of the race model with RMI violations, we introduced the relay model, a minimal extension that includes two races at sequential stages (Fig 1c). As a key feature, the relay model allows for cross-modal initiation in addition to statistical facilitation. In its most basic form, the model includes a single free parameter, the RT share, which partitions the overall RT between the processing stages. Our simulations and fits to benchmark data [4] demonstrate that the relay model captures the magnitude of the RSE, including RMI violations, and closely approximates the observed RT distribution (Fig 2). Moreover, we show that a two-stage architecture is sufficient to explain the RSE (Fig 3). We further show that the model captures decision dynamics under manipulations of temporal structure and signal intensity [37,38]. Specifically, the model captures the modulation of the RSE under asynchronous signal presentation (Fig 4) and under manipulations of signal strength (Fig 5). Together, these findings indicate that a two-stage relay configuration is both computationally parsimonious and descriptively powerful, providing an efficient and accurate framework for modelling multisensory decision-making.

Beyond statistical facilitation proposed by the classical race model [5], cross-modal initiation at stage 1 is the main new proposal of the relay model. While the relay model does not necessarily commit to a specific cognitive mechanism underlying the two stages, we propose that modality-specific signals at an initial gating stage race to initiate a second decision stage, which in turn triggers a behavioural response. The concept of gated accumulation is well-established in perceptual decision-making with unisensory signals, where gating selects relevant sensory input and determines the onset of evidence accumulation [e.g., 58,71,72]. Our work implies that the dynamics at the gating stage are not constant but are instead modulated by multisensory stimulation. As our model provides a clear handle on this stage, it may offer a valuable framework for investigating gating mechanisms and decision dynamics more generally.

While our work is currently limited to the modelling of behavioural data, the relay model framework can be linked to physiological evidence. For example, early event-related potential (ERP) components have been shown to predict the initiation of evidence accumulation [73,74], which could correspond to the initiation of the decision stage in the relay model. In multisensory studies, modulation of ERPs has been observed as early as 40–80 ms after stimulus onset [8,75,76], closely matching the first-stage durations predicted by the relay model (30–90 ms across the behavioural datasets analysed here [4,37,38]). Interestingly, some evidence suggests that early multisensory integration effects predominantly enhance neural activity in the non-dominant sensory modality [8]. Given auditory dominance (i.e., faster and more accurate responses), the relay model predicts that the auditory process would win the first race more frequently, allowing cross-modal initiation to benefit visual processing to a greater extent. Conversely, under visual dominance, the opposite pattern would be expected. These ERP markers therefore provide plausible neural correlates for the first model stage, linking the relay architecture to measurable brain dynamics. Future work could test this interpretation more directly through joint modelling of behaviour and neural recordings, potentially disentangling the first and second stages temporally and across sensory modalities.

As a technical note on the mathematical implementation of the relay model, we used a single RT share parameter for both modalities, partitioning processing times under the assumption that all components are IG distributed. Importantly, the model’s ability to account for the data and generate RMI violations does not depend on this specific choice. Because RMI violations are inherently driven by the mechanism of cross-modal initiation, they emerge under a variety of statistical assumptions. For example, a relay architecture with an exponential first stage and a Gaussian second stage also generates RMI violations. This flexibility extends to the model’s structural parameters: the assumption of a symmetrical RT share can be easily relaxed. In the presence of temporal asymmetries in peripheral sensory processing, the model can employ separate RT share parameters for each modality. While this would allow for more complex dynamics, our current simulations demonstrate that even a strict, single-parameter baseline is sufficient to capture the main decision dynamics underlying the RSE.

Conceptually, the relay model builds on the classical race model [5], with statistical facilitation as the primary driver of the RSE, but it is not the first framework to propose additional cross-modal interactions. For example, Mordkoff and Yantis [41] introduced an interactive architecture in which crosstalk occurs between parallel processing channels. Similarly, Nickerson [77] attributed facilitatory effects to enhanced response preparedness, whereby a fast auditory signal primes the system to respond more rapidly to a coincident visual stimulus. These frameworks provide important conceptual precedents for extending the classical race model and incorporating separate processing stages in models of multisensory decision-making.

Building on these foundations, the time-window-of-integration (TWIN) model provides an influential contemporary two-stage architecture [28,42]. The model accounts for multisensory facilitation by positing a peripheral sensory processing stage followed by a central pooling stage. While both the TWIN and the relay model share a race process at the initial stage, they differ significantly in their parameterisation and architectures, particularly at the second stage. To capture its complex processing dynamics, the TWIN model utilises a five-parameter architecture, with one free parameter specifically determining the magnitude of the RSE. Given its specific distributional assumptions, model fitting for the TWIN framework is typically performed at the level of mean RTs [78]. In contrast, the relay model maintains the classical race architecture at both stages, explaining the magnitude of the RSE largely through statistical facilitation and cross-modal initiation, both inherent in its structure. Furthermore, the relay model leverages a highly simplified architecture, using the RT share as a single free parameter beyond the fits to unisensory RT distributions, which allows it to accurately predict not only summary statistics but also the full shape of empirical RT distributions.

Furthermore, the relay model provides a mechanistically transparent alternative to other race-based accounts that add cross-modal interaction terms in addition to statistical facilitation as the main mechanism. For example, we included an ad hoc formulation of a shifted race model in the analysis of Miller’s benchmark data. While the model performed only slightly worse under synchronous conditions than the relay model, a key distinction emerges when considering SOA data, which requires the model to transition between unisensory and multisensory processing. In this case, the shifted race model fails without introducing additional free parameters. Another interactive race model was proposed by Otto and Mamassian [11], who introduced additive noise between parallel processes. While the addition of noise permits RMI violations, it is insufficient to reproduce the full shape of empirical RT distributions, requiring an additional correlation parameter to capture trial-by-trial dependencies [11]. Furthermore, because these parameters must be explicitly activated during multisensory conditions, the model lacks the intrinsic ability to transition between unisensory and multisensory regimes. In contrast, the relay model seamlessly transitions between regimes based solely on the temporal lag introduced by SOA manipulation: as the SOA increases, the likelihood of cross-modal initiation naturally diminishes, effectively reducing facilitation without requiring parameter adjustments.

The relay model can be easily adapted to other established manipulations of the redundant signals paradigm. For example, it may also be applied to redundant targets within the same sensory modality [15–19,79,80]. As the initiation stage relies on closely related processing pathways, cross-modal initiation is here expected to produce smaller facilitation effects. This prediction is consistent with the typically reduced RMI violations observed in such experiments. The relay model can also be naturally extended to experiments employing three redundant signals [9,20,61,81,82]. The typical finding is that that RSE for bimodal combinations is very similar to the experiments described in this manuscript; however, the benefit from adding a third signal is relatively smaller (both in terms of RSE and RMI violations). The relay model can be straightforwardly extended to three parallel processes at each stage. As the incremental benefit from statistical facilitation is reduced for additional racers, it directly predicts the observed empirical findings. The relay model therefore generalises naturally across task variants, providing new avenues for related research.

Beyond the redundant signals paradigm, the relay architecture also extends to focused attention tasks. In such tasks, participants are asked to respond to a signal in one modality, which in some trials is paired with a distractor signal in another modality. Although the task requires inhibiting the non-target modality, RTs are paradoxically faster when a distractor is presented alongside the target. The relay model can explain this finding by assuming that the initial gating stage is automatic and non-specific, such that both target and non-target signals are processed in parallel, racing to initiate the second stage and thereby enabling cross-modal initiation. The second stage, however, includes only a single processing pathway for the target signal, in accordance with the task demands. This mechanism produces a facilitation effect without generating erroneous responses to the distractor, aligning with foundational attention theories [e.g., 83] and computational frameworks like the Dual-Stage Two-Phase model [57].

One limitation of the current relay model is that it does not account for trial history effects, which have been shown to influence RTs in RSE experiments [11,39,84–89]. Unisensory responses are particularly sensitive to modality switches, often exhibiting RT costs following a switch that can rival the magnitude of the RSE itself. In contrast, multisensory responses tend to be less affected by the recent trial history (e.g., [84,85]), leading to an asymmetry that can modulate the RSE and exaggerate RMI violations. As such, any comprehensive model of multisensory integration should consider these sequential effects. In unisensory decision-making, recent models have begun to incorporate such dependencies. For instance, Caie et al. [72] introduced a gated accumulation model in which trial history influences the gating dynamics of sensory evidence accumulation. In future work, we propose extending the relay model in a similar direction by embedding history-dependent mechanisms into its initial stage. This extension would enable the model to capture trial history effects in unisensory conditions and generate testable predictions for multisensory responses without requiring additional parameter fitting.

In conclusion, the relay model provides a principled and computationally grounded framework for understanding the temporal dynamics of multisensory decision-making. By simply extending the classic race model to incorporate two races at sequential processing stages, it accounts for the full magnitude of the RSE, including RMI violations, while remaining mechanistically transparent. Its modular design allows for integration with broader decision-theoretic models, including those that incorporate gating, evidence accumulation, and trial history effects. As such, the relay model provides a new tool for linking behavioural findings to underlying neural and cognitive processes in multisensory integration.

## Methods

### Ethics Statement

The analysis carried out in this study involves model fitting of non-contentious anonymous secondary human data and did not involve any new participant data collection (see Data). Ethical approval for data reuse was given by the University Teaching and Research Ethics Committee (UTREC) at the University of St Andrews (0740 - PS-0740-852-2025).

### Data

The data presented in Fig 1b, along with the modelling results in Figs 2 and 3, are based on Experiment 1 from Miller [4]. In this experiment, 74 participants completed a redundant signals task involving three stimulus conditions: a visual stimulus (an asterisk at the centre of the screen), an auditory stimulus (a 780-Hz tone), or both stimuli simultaneously. Participants were instructed to press a key as quickly as possible upon detecting any stimulus. Each participant completed two blocks of 40 trials, with each block containing 10 trials per stimulus condition as well as 10 catch trials (no stimulus). For analysis, RTs for each condition within each block were rank-ordered and then averaged across blocks and participants [4,90]. This yielded three cumulative group RT distributions (auditory, visual, and audiovisual), each based on 1480 trials. As raw data are not available from the original publication, we extracted group-level data using graphical digitisation (Figure 1 in [4]). The figure was digitised, and the pixel-to-millisecond scaling was calibrated using a known 100 ms interval. RT values were then read directly from the scaled x-axis coordinates of the plotted points.

The data underlying Fig 4b are taken from Miller’s follow-up study [38]. Two participants completed the same redundant signals task as described above. Crucially, this study introduced temporal asynchronies between the auditory and visual stimuli. Audiovisual trials were divided into 11 conditions: a synchronous condition, five with the visual stimulus leading (SOAs from −33 to −167 ms), and five with the auditory stimulus leading (SOAs from 33 to 167 ms), all in 33 ms increments. Each participant completed 40 blocks of trials. Each block contained 170 trials, including 10 trials per stimulus conditions (13 conditions: auditory, visual, and 11 audio-visual SOAs) and 40 catch trials (no stimulus). We used data only from participant B.D., whose responses showed significant RSEs in 7 of the 11 SOA conditions. By contrast, participant K.Y.’s data showed significant RSEs in only three conditions, a finding that Miller described as “somewhat atypical in view of the consistency with which RSEs are obtained” [38]. Summary statistics were reported in the original publication for each condition, which included the mean, standard error, and median of RTs, as well as the magnitude of the RSE (Table 1 in [38]). The analysed data comprised 400 trials per condition.

The data underlying Fig 4c are based on Experiment 1 from Otto et al. [37]. Ten participants performed a redundant signals task involving a visual stimulus (random dot motion), an auditory stimulus (a 294-Hz tone), or both stimuli. For redundant conditions, the experiment featured five SOA conditions ranging in increments of 30 ms from -60 ms to +60 ms. The experiment was conducted in four blocks per participant. Following the original protocol, we removed outliers and retained 40 valid trials per block and condition. To combine data across the four blocks, we used Vincent averaging [90]. While Otto et al. [37] originally analysed group-level data, we analysed the data at the participant level. This approach provides a stricter test of the relay model, as it requires the model to capture the RT distributions of individual participants rather than a smoothed aggregate. Overall, the dataset comprised 1600 trials per condition.

The data underlying Fig 5 are based on Experiment 2 from Otto et al. [37]. Twenty participants performed a similar redundant signals task as described above. Here, the experiment manipulated auditory and visual signal strength in a 3x3 design. Auditory signals were weak (A_W_; 49.5 dB), medium (A_M_; 50.5 dB), and strong (A_S_; 53 dB). Visual signals varied in motion coherence at levels weak (V_W_; 20%), medium (V_M_; 30%), and strong (V_S_; 50%). For redundant conditions, the experiment tested all nine multisensory combinations (e.g., A_W_V_S_ or A_S_V_M_). Standard tests of the race model as well as our modelling approach require near-ceiling performance, implying that signals are detected on every trial. As the weak visual condition (V_W_) exhibited a high miss rate (>20%), we did not include conditions involving this level in our analysis. Data collection occurred over two sessions on separate days. As the dataset did not permit linking participant data across days, we followed the original protocol and treated each block as an independent sample. For each block, we removed outliers and retained the last 50 valid trials in each condition. Overall, the dataset comprised 2000 trials per condition. Data underlying all analyses can be accessed from [91].

### Unisensory RT distributions

Race models allow for parameter-free predictions of the RSE directly from the observed unisensory RT distributions. Basic predictions, such as those from Raab’s race model (probability summation, Eq 2) or the RMI (Eq 3), can be computed from empirical data without distributional assumptions (e.g., [92,93]). However, generating more complex predictions, such as those required by the relay model, requires assuming a particular RT distributional form.

For all simulations, we modelled unisensory RTs using the IG distribution (also known as Wald distribution; [94]). The IG distribution is well-suited for modelling non-negative, positively skewed data such as RTs [34,35]. Consequently, we assumed that unisensory RTs are IG-distributed with mean μ and shape parameter λ:


$$\begin{array}{c} RT\;\sim \;IG(\mu ,\;\lambda ) \end{array}$$ (5)

For the group-level RT distributions extracted from [4], we estimated the IG parameters for each unisensory condition separately: (μ_A_,λ_A_) for the auditory condition, and (μ_V_, λ_V_) for the visual condition. Parameters were optimised using MATLAB’s *fmincon* function, minimising the RMSE between the empirical and the simulated distributions at the 10 available quantiles.

For the SOA data extracted from [38], we estimated (μ_A_,λ_A_) and (μ_V_, λ_V_), using the method of moments approach [95]. Here, the fitting procedure minimised the RMSE between the observed and predicted descriptive statistics (mean and standard deviation). Although [38] reported standard errors, we converted these to standard deviations (using n = 400) to directly fit the standard deviation of the simulated distribution. The analytic mean was directly computed from μ and the standard deviation from $\sqrt{\mu^{\text{3}}/\lambda }$.

For both the SOA and the signal strength datasets from Otto et al. [37], unisensory parameter estimation was performed at the individual participant level using Quantile Maximum Probability Estimation (QMPE; [63,64]). For each condition, we computed five quantiles from the raw RT distributions, defining six bins. Parameters were optimised by minimising the negative log-likelihood (NLL) of the observed bin counts using MATLAB’s *fmincon* function. For the SOA dataset [37], IG parameters (μ, λ) were fitted independently for auditory and visual conditions, resulting in four unisensory parameters per participant. For the signal strength dataset [37], IG parameters were fitted independently for each intensity level (five conditions: A_S_, A_M_, A_W_, V_S_, and V_M_), resulting in ten unisensory parameters per participant.

Table 1 reports the best-fitting IG parameters for all unisensory conditions. These parameters were held fixed and used to constrain all subsequent modelling of multisensory conditions, including race model predictions and relay model simulations.

### Stage-based partitioning of unisensory RTs

The relay model assumes that unisensory RTs are composed of two (or more) sequential processing stages (Fig 1c). Accordingly, the total RT can be expressed as


$$\begin{array}{c} RT=\;\sum_{i=\text{1}}^{n}T_{i} \end{array}$$ (6)

where i is the index of n stages.

To estimate the contribution of these (latent) stages, we implemented an approach that partitions the overall RT into stage-specific processing times. This approach relies on a key property of inverse Gaussian (IG) distributions: under specific conditions, the sum of IG-distributed random variables is itself IG-distributed. Specifically, if


$$\begin{array}{c} T_{i}\;\sim \;IG\left(\mu w_{i},\;\lambda \overset{\text{2}}{\underset{i}{w}}\right)\;with\;w_{i}>\text{0}, \end{array}$$ (7)

describes the processing time at (latent) stages, then the total unisensory RT remains IG-distributed with mean μ and shape parameter λ (Eq 5). This property arises because the IG parameters are scaled with the weights $w_{i}$, such that the convolution preserves the original distributional form. While typically used as a summation rule, we exploit this property in reverse: it allows us to decompose a single, empirically fitted IG distribution into (latent) constituent stages. As a result, the unisensory RT distributions, which are overall described by IG distributions (Table 1), can equivalently be expressed as the sum of two or more (latent) IG-distributed processing stages, with the mean and variance partitioned via the weights $w_{i}$.

For model fitting and simulation purposes in the multisensory context, we imposed two constraints on the partitioning approach. First, we used normalised weights $w_{i}$ that sum to one:


$$\begin{array}{c} \sum_{i=\text{1}}^{n}w_{i}=\text{1} \end{array}$$ (8)

Thus, weights $w_{i}$ can be interpreted as the proportion of the total RT contributed by a specific stage (in terms of both mean and variance), which we refer to as the RT share (Fig 2a). For example, $w_{\text{1}}=\text{0}.\text{2}$ indicates that stage 1 accounts for 20% of the total RT. Stage 2 would then account for the remaining 80%.

Second, for simplicity, we assumed that the same RT share configuration applies to both audition and vision. For example, if the RT share at the first auditory stage is 20%, we assumed the same RT share for the corresponding visual stage. This simplification facilitates model fitting and interpretation but can be relaxed in future experiments aimed at isolating modality-specific stage dynamics.

### Relay model

The basic relay model consists of two sequential race units (Fig 1c). Using the partitioning approach established above, for stage i, let the auditory and visual processing times be distributed as:


$$\begin{array}{c} T_{Ai}\;\sim \;IG\left(\mu_{A}w_{i},\;\lambda_{A}\overset{\text{2}}{\underset{i}{w}}\right),\;T_{Vi}\;\sim \;IG\left(\mu_{V}w_{i},\;\lambda_{V}\overset{\text{2}}{\underset{i}{w}}\right) \end{array}$$ (9)

where $w_{i}$ represents the RT share of stage i. Because this partitioning preserves the IG form, the corresponding probability density functions (PDFs) and cumulative distribution functions (CDFs) can be expressed analytically. We denote the PDFs as $f_{Ai}(t)$ and $f_{Vi}(t)$, and the CDFs as $F_{Ai}(t)$ and $F_{Vi}(t)$, respectively.

In the multisensory condition, stage i is completed as soon as either modality finishes processing. The duration of stage i is therefore given by:


$$\begin{array}{c} T_{AVi}=min\left(T_{Ai},T_{Vi}\right) \end{array}$$ (10)

The corresponding CDF, denoted $F_{AVi}(t)$, can be computed analogously to Raab’s race model (Eq 2), assuming statistical independence between auditory and visual processes within each stage. It can be analogously expressed as


$$\begin{array}{c} F_{AVi}(t)=\text{1}-S_{Ai}(t)\times S_{Vi}(t) \end{array}$$ (11)

where $S_{Ai}(t)=\text{1}-F_{Ai}(t)$ denotes the survival function. The corresponding PDF, denoted $f_{AVi}(t)$, is given by:


$$\begin{array}{c} f_{AVi}(t)=f_{Ai}(t)S_{Vi}(t)+f_{Vi}(t)S_{Ai}(t) \end{array}$$ (12)

As the relay model assumes cross-modal initiation, the total multisensory ${RT}_{AV}$ is given by the sum of the durations of the sequential stages (Eq 4). For analytical tractability, we assume independence between stages, such that the durations of successive stages are treated as independent random variables. To compute the CDF of ${RT}_{AV}$, we convolved the CDF of the first stage ($F_{AV\text{1}}$) with the PDF of the second stage ($f_{AV\text{2}}$):


$$\begin{array}{c} F_{AV}(t)=\int_{\text{0}}^{\infty }F_{AV\text{1}}(t-x)f_{AV\text{2}}(x)dx \end{array}$$ (13)

For simulations shown in Fig 3, we extended the relay model to more than two stages. The overall CDF for a multi-stage relay model was computed iteratively: starting with the CDF of the first stage, we successively convolved it with the PDFs of the additional stages. Each iteration follows the same computation as in Eq 13.

To provide a simple alternative to the relay model, we also fitted a shifted race model, a variant of the race model in which the predicted RT is shifted by a constant δ, acting as a single free parameter. The corresponding CDF is given by:


$$\begin{array}{c} F_{AV}\left(t|\delta \right)=\text{1}-[S_{A}(t+\delta )\times S_{V}(t+\delta )] \end{array}$$ (14)

where $F_{AV}\left(t|\delta \right)$ denotes the probability of a multisensory response by time *t* given a shift δ, and $S_{m}(t+\delta )$ is the survivor function of modali*t*y m evaluated at the shifted time.

To account for SOA manipulations [37,38], we extended the two-stage relay model to incorporate onset lags. We defined the lag, τ, as the time difference between the onsets of the leading and the lagging signal. This lag is not a free model parameter, instead it is directly determined by the experimentally defined SOA condition. Adding τ can affect both relay model stages ($T_{AV\text{1}},T_{AV\text{2}}$). At stage 1, τ introduces a temporal disadvantage for the lagging modality. For example, if the visual signal is delayed by τ, the duration of stage 1 is given by:


$$\begin{array}{c} T_{AV\text{1}}=min\left(T_{A\text{1}},T_{V\text{1}}+\tau \right) \end{array}$$ (15)

As described above, the first stage initiates the second stage at time $T_{AV\text{1}}$. Intuitively, if stage 2 begins before the delayed signal is presented ($T_{AV\text{1}}<\tau$), that modality enters the race with a residual temporal disadvantage (equal to $\tau -T_{AV\text{1}}$). Continuing with the example of a delayed visual signal, the duration of stage 2 is therefore given by:


$$\begin{array}{c} T_{AV\text{2}}=min\left(T_{A\text{2}},T_{V\text{2}}+\text{max}(\text{0},\;\tau -T_{AV\text{1}})\right) \end{array}$$ (16)

To describe the effect of temporal delays between signals, we divided the model predictions into two scenarios depending on the time u at which stage 2 is initiated. In the first, early initiation scenario, stage 2 begins before the lagging signal has been presented (u<τ). In this case, stage 1 is driven entirely by the leading signal. For example, if the visual signal lags by τ, the contribution of the early initiation scenario to the overall model CDF is:


$$\begin{array}{c} \Phi_{Early}(t)=\int_{\text{0}}^{\text{min}(t,\tau )}\underset{\text{Stage }\text{1}}{\underbrace{f_{A\text{1}}(u)}}\underset{\text{Stage }\text{2}}{\underbrace{\left[\text{1}-S_{A\text{2}}(t-u)S_{V\text{2}}(t-\tau )\right]}}du \end{array}$$ (17)

Here, $f_{A\text{1}}(u)$ denotes the probability density of the stage 1 processing time of the leading (auditory) modality. The stage 2 term follows Eq 11 but is adapted to reflect that visual processing can only begin after the lag τ has elapsed. In other words, stage 2 begins immediately for the leading modality (after time u), whereas the lagging modality can only start after its signal onset (at time τ), resulting in a residual temporal disadvantage.

In the second, late initiation scenario, stage 2 begins after the lagging signal has been presented (u≥τ). In this case, stage 1 is driven by a race between both modalities. Continuing with the example where the visual signal lags by τ, the contribution of the late initiation scenario to the overall model CDF is:


$$\begin{array}{c} \Phi_{Late}(t)=\int_{\tau }^{\text{t}}\underset{\text{Stage }\text{1}}{\underbrace{g(u)}}\underset{\text{Stage }\text{2}}{\underbrace{\left[\text{1}-S_{A\text{2}}(t-u)S_{V\text{2}}(t-u)\right]}}du \end{array}$$ (18)

Here, g(u) denotes the probability density of the stage 1 completion time under a race between both modalities, which can be computed following Eq 12, adapted to account for the lag τ in the visual modality:


$$\begin{array}{c} g(u)=f_{A\text{1}}(u)S_{V\text{1}}(u-\tau )+f_{V\text{1}}(u-\tau )S_{A\text{1}}(u) \end{array}$$ (19)

Regarding the stage 2 term, because both signals are present at time u, processing starts immediately for both modalities, and Eq 11 applies directly. The total predicted CDF is then given by the sum of the two components:


$$\begin{array}{c} {\text{F}}_{AV}(t)=\Phi_{Early}(t)+\Phi_{Late}(t) \end{array}$$ (20)

### RSE and RMI violations

To inspect the key features of the predictions derived from the relay model, we measured the RSE as well as violations of the RMI (Figs 2 and 3). To quantify these effects, we used geometric measures on the level of CDFs analogous to approaches described elsewhere [93,96].

### Model fitting

In addition to model simulations, we fitted the relay model to empirical data from [4,37,38]. As outlined above, the relay model was constrained by the unisensory RT distributions estimated for each dataset (Table 1). To account for the observed multisensory RTs, the model included a single free parameter: the RT share of stage 1, constrained to the interval [0, 0.5].

To fit the relay model to the multisensory RT distributions from [4], we compared empirical quantiles to the corresponding quantiles predicted by the relay model CDF (Eq 13). The RMSE between the empirical and model CDF was minimised by adjusting the RT share parameter. Optimisation was conducted using MATLAB’s *fminbnd* function. All fitting procedures, including parameter recovery simulations, can be accessed from [91].

Similarly, we fitted the shifted race model to the same data (Eq 14). Here, the RMSE between the empirical and model CDF was minimised by adjusting the shift parameter δ. The parameter search was constrained to ensure physical plausibility. The upper bound was fixed at 500 ms, while the lower bound was dynamically set for each condition as to ensure that the shifted times remained strictly positive. Optimisation was performed using MATLAB’s *fmincon* function.

To fit the relay model to the multisensory RTs from [38], we used the relay model adapted for temporal asynchronies (Eq 20). Miller [38] defined the RSE as the difference in mean RT between the faster unisensory and the multisensory condition. To generate model predictions, we computed representative RT distributions for each condition and SOA by evaluating the inverse CDF (quantile function) from Equation 20 at 200 evenly spaced probability values. Using these predicted RTs, we calculated the RSE analogously to Miller [38]. We estimated the optimal RT share by minimising the RMSE between the empirical and predicted RSEs across the 11 SOA conditions. Optimisation was performed using MATLAB’s *fminbnd* function.

The RT share parameter was fit to the multisensory conditions in both the SOA and signal strength datasets from Otto et al. [37] using QMPE [63,64]. For the SOA dataset, we fitted the RT share parameter by minimising the joint NLL summed across all six SOA conditions (-60, -30, 0, 30, and 60 ms). This approach ensures that a single multisensory RT share accounts for the entire range of temporal asynchronies. For the signal strength dataset, we applied a stricter test of model generalisability. To test the model’s predictive validity, we fitted the RT share parameter only to the congruent medium-intensity condition (A_M_V_M_). This fitted RT share was then used to predict RTs for remaining five signal strength combinations (e.g., A_S_V_S_ or A_W_V_M_) without further optimisation, effectively treating these conditions as out-of-sample tests.
